# Use of plant stanol ester margarine among persons with and without cardiovascular disease: Early phases of the adoption of a functional food in Finland

**DOI:** 10.1186/1475-2891-4-20

**Published:** 2005-06-01

**Authors:** Meri Simojoki, Riitta Luoto, Antti Uutela, Hannu Rita, John D Boice, Joseph K McLaughlin, Pekka Puska

**Affiliations:** 1 Department of Epidemiology and Health Promotion, National Public Health Institute, Helsinki, Finland; 2 The UKK Institute for Health Promotion, Tampere, Finland; 3 Department of Forest Resource Management, University of Helsinki, Finland; 4 International Epidemiology Institute, Rockville, Maryland, USA; 5 Department of Medicine, Vanderbilt University Medical Center and Vanderbilt-Ingram Cancer Center, Nashville TN, USA

**Keywords:** Plant stanol, margarine, life-style, cardiovascular disease, Finland

## Abstract

**Background:**

The plant stanol ester margarine Benecol^® ^is a functional food that has been shown to lower effectively serum total and LDL-cholesterol. The purpose of this post-marketing study is to characterize users of plant stanol ester margarine with and without cardiovascular disease.

**Methods:**

A cohort of plant stanol ester margarine users was established based on a compilation of 15 surveys conducted by the National Public Health Institute in Finland between 1996–2000. There were 29 772 subjects aged 35–84 years in the cohort. The users of plant stanol ester margarine were identified by the type of bread spread used.

**Results:**

The plant stanol ester margarine was used as bread spread by 1332 (4.5%) subjects. Almost half (46%) of the users reported a history of cardiovascular disease. Persons with cardiovascular disease were more likely to use plant stanol ester margarine (8%) than persons without cardiovascular disease (3%). Users with and without cardiovascular disease seemed to share similar characteristics.

In particular, they were elderly people with otherwise healthy life-styles and diet. They were less likely smokers, more likely physically active and less likely obese than nonusers. The users reported being in good or average health in general and having used cholesterol-lowering drugs.

**Conclusion:**

Plant stanol ester margarine seems to be used by persons for whom it was designed and in a way it was meant: as part of efforts for cardiovascular disease risk reduction.

## Introduction

Functional foods are becoming increasingly available to consumers worldwide. A food can be regarded as "functional" if it has beneficial effects on one or more target functions in the body, beyond obvious nutritional effects, that are related to either an improved state of health and well-being and/or a reduction of disease risk [1]. As the health effects of long-term use of functional foods are often unknown, there is a need for post-marketing research [2-4].

The plant stanol ester margarine Benecol^® ^(Raisio Group, Raisio, Finland), launched in Finland in 1995, is an example of a functional food that has been shown to lower effectively serum total and LDL-cholesterol levels in several interventions [5,6]. Dietary plant sterols, especially sitostanol, reduce serum cholesterol by inhibiting cholesterol absorption. The present source of plant sterol is extraction from a byproduct inthe refining of vegetable oils or from the oil obtained from pinewoodpulp in papermaking. A tenth of the amount of plant sterols found in these plant sterol margarines occurs naturally in a normal diet. Use of plant sterol ester margarine is recommended, as part of a healthy diet, by the Finnish Society for Internal Medicine for the primary and secondary prevention of coronary heart disease in Finland [7].

Elevated blood cholesterol is one of the main causal risk factors for coronary heart disease [8]. Other factors that affect coronary heart disease include socio-demographic and life-style characteristics such as age, gender, education, region, body-mass index, smoking, physical activity and diet [9]. As many of these factors relate to life-style, the risk can be modified to great extent by changes in health behavior. Indeed, the North Karelia Project in Finland revealed that population rates of cardiovascular diseases can be influenced remarkably by changes in people's dietary habits [8].

The National Public Health Institute in Finland has monitored health behaviors and chronic disease risk factors in the Finnish population regularly since 1972. Two survey systems are especially relevant for the present study: the Finnish Adult Health Behavior Survey [10] and the Chronic Disease Risk Factor Survey (Finrisk) [11]. In these surveys, random samples of the Finnish population are drawn and risk factors are assessed by a self-administered questionnaire and direct measurements. Dietary habits, smoking and physical activity are of special interest in the monitoring. The surveys conducted in 1996 or later include a question on plant stanol ester margarine use.

The health effects, both beneficial and adverse, of long-term use of the plant stanol ester margarine have not been studied. Therefore, a post-marketing study was started at the National Public Health Institute in Finland in 1998 [12]. The study includes several sub-studies where existing survey data as well as health registries in Finland are analyzed. The data on the adoption of the functional food during early post-marketing phase is unique and of importance in relation also to other products of the kind and to the efficacy of dietary counseling.

Our previous study revealed that users of plant stanol ester margarine are elderly people (mean age 59 years), about half of whom have been diagnosed with some form of cardiovascular disease [12]. The purpose of this study is to characterize users of plant stanol ester margarine with and without cardiovascular disease at an early post-marketing phase. The characteristics of interest are socioeconomic background, life-style factors, diet and medication. This information is needed when assessing the efficacy of dietary counseling and the possible health effects of the long-term use of plant stanol ester margarine.

## Methods

Fifteen independent health surveys conducted by the National Public Health Institute in Finland between 1996 and 2000 were combined into study cohort (Table 1 in Additional File 1). The main surveys were the annual nationwide Finnish Adult Health Behavior Survey and the Chronic Disease Risk Factor Survey (Finrisk) carried out in five areas of Finland. In each survey, sampling was either entirely random or stratified random with respect to age and geographical area.

In each survey, the subjects completed mailed questionnaires concerning their health and life-style and returned them by mail or in person. In the Finrisk Survey, clinical stage with health examination was included. In the Finrisk Senior Survey, elderly participants aged 65–74 years, who were not able to come to the health examination, were interviewed and examined at their home. The participation rates in the surveys varied between 64–79%.

The users of plant stanol ester margarine were identified on the basis of the following question present in each survey questionnaire: "What type of fat do you usually use on bread?" Choices were no fat at all, low-fat spread, soft margarine, butter-oil mixture, butter, and plant stanol ester margarine Benecol^®^. The plant stanol ester margarine Benecol^® ^was the only plant sterol margarine available for consumers in Finland during the time the health surveys were conducted.

Subjects who reported myocardial infarction, stroke, hypertension, heart failure, angina pectoris or intermittent claudication were classified as having cardiovascular disease. Subjects who did not report any of these conditions were classified as not having cardiovascular disease.

Overall, 42 692 questionnaires were returned. When duplicate respondents were identified, only the first response was retained, thereby reducing the size of the cohort to 42 406 subjects. Because cardiovascular diseases and the use of plant stanol ester margarine are infrequent in younger age groups, we further excluded respondents aged 15–34 years from the analysis. The final study population consisted of 29 772 subjects aged 35–84 years, 14 262 men and 15 510 women.

Unadjusted prevalence percentages among users with and without cardiovascular disease were computed for age, sex, socioeconomic background, life-style, body mass index, self-perceived health, diet and medication. Odds ratios and 95% confidence limits adjusted for age were calculated using logistic regression models to compare users and nonusers of plant stanol ester margarine. Subjects with and without cardiovascular disease were combined in odds ratio calculations. Data management and statistical analysis were performed using the SAS System (version 6.12) [13].

## Results

Overall, the plant stanol ester margarine was used as the bread spread of choice by 1332 (4.5%) subjects. Almost half (46%) of the users and one fourth (25%) of the nonusers reported a history of cardiovascular disease. Table 2 (in Additional File 2)presents the distribution of cardiovascular diseases among users and nonusers of plant stanol ester margarine.

Among subjects with cardiovascular disease, the proportion of users of plant stanol ester margarine was 8%, whereas the proportion among subjects without the disease was 3% (Table 3 in Additional File 3). In general, users with and without cardiovascular disease shared similar characteristics. The use of plant stanol ester margarine was most common among subjects aged 65–74 years. Men were slightly more likely to use plant stanol ester margarine than women. Urban and married people with higher levels of education were represented more among the users than nonusers of plant stanol ester margarine.

Among the users of plant stanol ester margarine, there were less smokers and more physically active people as well as less of those with high levels of stress compared with nonusers (Table 4 in Additional File 4). The users were also less often obese (defined as body mass index over 30 kg/m^2^).

Self-perceived health was good or average among users of plant stanol ester margarine (Table 5 in Additional File 5). Users of plant stanol ester margarine consumed higher amounts of alcohol compared with nonusers. Substantially greater proportion of users also consumed a healthy diet compared with nonusers.

Users of plant stanol ester margarine used cholesterol-lowering drugs more commonly than nonusers (Table 6 in Additional File 6). The use of hypertensive drugs, diabetic drugs and acetylsalicylic acid for acute myocardial infarction (AMI) prevention, as well as cardiovascular disease drugs, were also more common among users than nonusers of plant stanol ester margarine.

Some continuous variables were evaluated separately for users with and without cardiovascular disease. The mean age was 63 years among the users with cardiovascular disease and 57 years among the users without the disease. The amounts of plant stanol ester margarine on bread were almost the same among users with and without cardiovascular disease: 21 and 20 g per day, respectively.

Blood cholesterol had been measured among 82% and 64%, elevated blood cholesterol had been diagnosed among 85% and 72%, and dietary counseling for lowering of blood cholesterol had been received by 83% and 71%, of users with and without cardiovascular disease, respectively. Among nonusers, the respective proportions were substantially lower. The percentages for blood cholesterol measurement, elevated blood cholesterol, and dietary counseling were 58% and 28%, 48% and 25%, and 52% and 31%, for nonusers with and without cardiovascular disease, respectively.

## Discussion

The users of plant stanol ester margarine are a self-selected group of persons who have taken an active interest in their health. They use plant stanol ester margarine as part of a generally healthy life-style and diet. Nevertheless, they commonly have a history of cardiovascular disease or are at risk to have it. Thus plant stanol ester margarine seems to be used by persons for whom it was designed and in a way it was meant: as part of efforts for cardiovascular disease risk reduction.

Users of plant stanol ester margarine appear in this early post-marketing adoption phase to be elderly people. They reported generally good or average health status and took more commonly cholesterol-lowering drugs as compared with nonusers. Overall, users with cardiovascular disease seemed to share similar characteristics compared with users without cardiovascular disease, although users with cardiovascular disease tended to be slightly older.

As the plant stanol ester margarine has been recommended in Finland for the primary and secondary prevention of coronary heart disease [7], we assume that the users with cardiovascular disease attempt to control elevated cholesterol levels and prevent the progress of the disease. Users of plant stanol ester margarine who do not have cardiovascular disease probably also aim to control cholesterol levels and thus prevent cardiovascular disease. Unfortunately, we were unable to directly evaluate this issue, since data on indication for use of plant stanol ester margarine was not collected.

However, the plant stanol ester margarine users both with and without cardiovascular disease obviously had more cholesterol problems than nonusers as they reported more of elevated blood cholesterol and dietary counseling for lowering of blood cholesterol. Furthermore, as the price of the plant stanol ester margarine is 3–5 times higher than other margarines, its use is likely based on clearly perceived need, i.e. the control of blood cholesterol levels.

The use of plant stanol ester margarine may also result from "practical reasons": if one spouse uses it, the other may be more likely to use it too. Previous studies show spouse concordance for physiological and behavioral indicators such as plasma cholesterol and triglyceride, blood pressure, diet, body mass index, smoking and physical exercise [14-17]. The similarity in the characteristics of plant stanol ester margarine users with and without cardiovascular disease might well be explained by family reasons.

Our information on the use of plant stanol ester margarine was based on a single question concerning the type of bread spread usually used. Although we have not validated the question against real use, we assume that the question reliably measures current use and that conclusions can be drawn concerning the characteristics of the users. Information concerning the quantity of the plant stanol ester margarine use was obtained from the Finrisk survey and was shown to be 20 g per day, on an average. A picture from a portion size picture booklet describing the amount of spread on bread was used to improve the validity of the estimation [18]. The quantity should be 20–25 g per day to achieve an optimal cholesterol-lowering effect [6,7], but it is clear from our data that higher levels of use are common.

The age and geographic distributions of the study cohort of 42 406 persons differ slightly from the general Finnish population. The proportion of persons aged 55–64 years is greater (24% versus 20%) and 65–84 years smaller (19% versus 24%) than in the general population. The proportion of persons living in eastern Finland is greater (20% versus 11%) and southern Finland smaller (30% versus 40%) compared with the general population. As the proportion of users is high among less represented groups, i.e. persons aged 65–74 years and those living in southern and thus urban areas of Finland, our cohort probably underestimates the frequency of the use of plant stanol ester margarine in the Finnish population.

Functional foods are usually carefully examined with respect to their health effects and safety before they come to market. These studies often have limitations such as short length of follow-up. Also effects in certain population or patient subgroups might be unknown due to small sample size. Therefore there is a need for post-marketing surveillance. Until now only few post-marketing studies concerning functional foods have been carried out namely with a food additive sweetener aspartame [19] and a non-energy fat substitute olestra [20] as the most notable exceptions.

The LDL-cholesterol reducing effect of plant sterols and stanols may result in reduced heart disease rates. However, they appear to somewhat lower lipid-standardized concentrations of the plasma carotenoids [2,5]. Also the health effects of high daily amounts of plant sterols and stanols are unknown. In our future follow-up studies, we will evaluate possible health effects, both beneficial and adverse, of the long-term use of the plant stanol ester margarine by linking available Finnish health outcome registries to the study cohort [12].

## Competing interests

MS in Finland and JDB and JKM in Maryland, USA, have received funding and/or salary from McNeil Consumer Healthcare Company and Raisio Group, Raisio, Finland. However, neither of these organizations is financing publication of this manuscript. None of the authors hold currently any stocks or shares in McNeil Consumer Healthcare Company or Raisio Group.

## Authors' contributions

MS, RL and AU conceived the study, MS performed the statistical analysis. JDB and JKM participated to the collection of the cohort, funding, statistical analysis and drafting of the manuscript. HR helped in statistical issues. PP participated in general supervision of the research group, drafting the manuscript and collecting the data base. All authors have read and approved the manuscript.

## Supplementary Material

Additional File 1 Population surveys in Finland assembled for plant stanol ester margarine evaluation (Table 1). Click here for file

Additional File 2 Self-reported cardiovascular diseases among 35–84 year-old users and nonusers of plant stanol ester margarine (Table 2) Click here for file

Additional File 3 Age, sex, and socioeconomic background among 35–84 year-old users and nonusers of plant stanol ester margarine (Table 3) Click here for file

Additional File 4 Life-style characteristics and body mass index among 35–84 year-old users and nonusers of plant stanol ester margarine (Table 4) Click here for file

Additional File 5 Self-perceived health and diet among 35–84 year-old users and nonusers of plant stanol ester margarine. (Table 5) Click here for file

Additional File 6 Medication among 35–84 year-old users and nonusers of plant stanol ester margarine (Table 6) Click here for file
