# Supplementary material for: Use of plant stanol ester margarine among persons with and without cardiovascular disease: Early phases of the adoption of a functional food in Finland
Source: Nutr J. 2005 Jun 1;4:20. doi: 10.1186/1475-2891-4-20 (PMC1177987; doi:10.1186/1475-2891-4-20)
Supplement: Additional File 1 — Population surveys in Finland assembled for plant stanol ester margarine evaluation (Table 1). [file 1475-2891-4-20-S1.rtf]

Table 1. Population surveys in Finland assembled for plant stanol ester margarine evaluation

Survey	Area	Sample	Total
N	Men
N	Women
N	Response rate %	Age
Years	
FAHBS 1996 a	Entire country	Random	3597	1669	1928	72	15-64	
FAHBS 1997 a	Entire country	Random	3516	1588	1928	70	15-64	
FAHBS 1998 a	Entire country	Random	3505	1689	1816	70	15-64	
FAHBS 1999 a	Entire country	Random	3371	1538	1833	68	15-64	
FAHBS 2000 a	Entire country	Random	3468	1569	1899	70	15-64	
North Karelia Survey 1996	North Karelia	Random	1259	624	635	68	15-64	
Kainuu Area Survey 1996 	Kainuu	Random	1316	668	648	68	15-64	
Several Municipalities Survey 1996	Heinola, Kerava, Lapinlahti, Pori, Tampere, Luoto, Kajaani	Random	1384	665	719	66	15-64	
Turku Survey 1996	Turku 	Stratified random	1867	797	1070	67	15-64	
Turku Survey 1997	Turku	Stratified random	1788	784	1004	64	15-64	
Recession Survey 1997	Entire country	Random	5494	2409	3085	74	22-72	
Elderly Health Behavior Survey 1997 	Entire country	Stratified random	1886	953	933	79	65-84	
Elderly Health Behavior Survey 1999 	Entire country	Stratified random	1794	858	936	75	65-84	
Finrisk 1997 Survey b

	Helsinki /Vantaa, Turku/Loimaa, Kuopio, Oulu, North Karelia	Stratified random	8272	4135	4137	72	25-74
	
Finrisk 1997 Senior Non-respondent Survey c 	Helsinki/Vantaa, North Karelia	-	175	119	56	- 	65-74	
Combined cohort d			42 406	19 946	22 460	64-79	15-84	
a Finnish Adult Health Behavior Survey.
b Chronic Disease Risk Factor Survey.
c Study among elderly non-respondents in the Chronic Disease Risk Factor Survey, 175 out of 387 non-respondents interviewed at their home.
d Only the first survey results from each respondent included. 
