# Supplementary material for: Use of plant stanol ester margarine among persons with and without cardiovascular disease: Early phases of the adoption of a functional food in Finland
Source: Nutr J. 2005 Jun 1;4:20. doi: 10.1186/1475-2891-4-20 (PMC1177987; doi:10.1186/1475-2891-4-20)
Supplement: Additional File 2 — Self-reported cardiovascular diseases among 35–84 year-old users and nonusers of plant stanol ester margarine (Table 2) [file 1475-2891-4-20-S2.RTF]

Table 2. Self-reported cardiovascular diseases among 35-84 year-old users and nonusers of plant stanol ester margarine. RR=relative risk of disease among users compared to non-users.

Reported cardiovascular disease	Users of plant stanol ester margarine	Nonusers of plant stanol ester margarine		
	Yes	No	Total	Yes	No	Total	RR	
	N	%	N	%	N	%	N	%	N	%	N	%		
Myocardial infarction ever a	41	11	321	89	362	100	237	4	6038	96	6275	100	3.0	
Myocardial infarction during past 12 months	28	3	933	97	961	100	277	1	21 402	99	21 679	100	2.3	
Stroke ever a	22	6	341	94	363	100	155	2	6112	98	6267	100	2.5	
Hypertension during past 12 months	463	35	857	65	1320	100	5709	20	22 188	80	27 897	100	1.7	
Heart failure during past 12 months	72	5	1244	95	1316	100	1110	4	26 734	96	27 844	100	1.4	
Angina pectoris during past 12 months	199	15	1119	85	1318	100	1685	6	26 168	94	27 853	100	2.6	
Intermittent claudication during past 12 months b	7	6	102	94	109	100	47	4	1076	96	1123	100	1.5	
At least one cardiovascular disease 	611	46	721	54	1332	100	7141	25	20 951	75	28 092	100	1.8	
a Only Finrisk 1997 Survey. 
b Only Finrisk 1997 Senior Non-respondent Survey.
