# Supplementary material for: Use of plant stanol ester margarine among persons with and without cardiovascular disease: Early phases of the adoption of a functional food in Finland
Source: Nutr J. 2005 Jun 1;4:20. doi: 10.1186/1475-2891-4-20 (PMC1177987; doi:10.1186/1475-2891-4-20)
Supplement: Additional File 3 — Age, sex, and socioeconomic background among 35–84 year-old users and nonusers of plant stanol ester margarine (Table 3) [file 1475-2891-4-20-S3.rtf]

Table 3. Age, sex, and socioeconomic background among 35-84 year-old users and nonusers of plant stanol ester margarine.

Characteristics	Subjects with cardiovascular disease	Subjects without cardiovascular disease	Users versus nonusers a	
	Users	Nonusers	Total	User	Users	Nonusers	Total	User		
	N	N	N	% b	N	N	N	% b	OR	CL (95%)	
10-year age group											
35-44	25	697	722	3	68	7323	7391	1	1.00		
45-54	113	1686	1799	6	236	6754	6990	3	2.24	1.66-3.03	
55-64	210	2263	2473	8	243	4341	4584	5	2.32	1.48-3.64	
65-74	190	1546	1736	11	144	1856	2000	7	2.08	1.11-3.91	
75-84	73	949	1022	7	30	677	707	4	0.84	0.36-1.92	
Total	611	7141	7752	8	721	20 951	21 672	3			
Sex											
Men	339	3658	3997	8	367	9718	10 085	4	1.19	1.07-1.34	
Women	272	3483	3755	7	354	11 233	11 587	3	1.00		
Total	611	7141	7752	8	721	20 951	21 672	3			
Education c											
Low	133	2190	2323	6	124	5474	5598	2	1.00		
Middle	174	2328	2502	7	199	6741	6940	3	1.26	1.07-1.48	
High	295	2398	2693	11	378	8264	8642	4	1.93	1.67-2.24	
Total	602	6916	7518	8	701	20 479	21 180	3			
Marital status											
Married d	451	4917	5368	8	564	15 372	15 936	4	1.52	1.34-1.74	
Single	160	2193	2353	7	154	5488	5642	3	1.00		
Total	611	7110	7721	8	718	20 860	21 578	3			
Urbanization											
Capital area	130	1099	1229	11	163	3267	3430	5	1.74	1.48-2.04	
Big cities	155	1755	1910	8	202	5897	6099	3	1.34	1.15-1.56	
Small cities	156	1883	2039	8	183	5109	5292	3	1.27	1.09-1.49	
Rural area	170	2404	2574	7	173	6678	6851	3	1.00		
Total	611	7141	7752	8	721	20 951	21 672	3			
a Odds ratio (OR) and 95% confidence limits (CL) adjusted for age, subjects with and without cardiovascular disease combined.
b % of total in the category.
c Educational years of each birth year have been divided into tertiles.
d Those in common-law marriage included.
