# Supplementary material for: Use of plant stanol ester margarine among persons with and without cardiovascular disease: Early phases of the adoption of a functional food in Finland
Source: Nutr J. 2005 Jun 1;4:20. doi: 10.1186/1475-2891-4-20 (PMC1177987; doi:10.1186/1475-2891-4-20)
Supplement: Additional File 4 — Life-style characteristics and body mass index among 35–84 year-old users and nonusers of plant stanol ester margarine (Table 4) [file 1475-2891-4-20-S4.rtf]

Table 4. Life-style characteristics and body mass index among 35-84 year-old users and nonusers of plant stanol ester margarine.

Characteristics	Subjects with cardiovascular disease	Subjects without cardiovascular disease	Users versus nonusers a	
	Users	Nonusers	Total	User	Users	Nonusers	Total	User		
	N	N	N	% b	N	N	N	% b	OR	CL (95%)	
Smoking											
Non-smoker	328	3548	3876	8	416	10 171	10 587	4	1.00		
Quitter	206	2048	2254	9	186	4679	4865	4	1.07	0.94-1.21	
Smoker	53	1224	1277	4	92	5517	5609	2	0.51	0.42-0.61	
Total	587	6820	7407	8	694	20 367	21 061	3			
Leisure-time physical activity										
	
Often	293	3154	3447	9	428	11 237	11 665	4	1.84	1.48-2.28	
Some	87	1202	1289	7	140	5282	5422	3	1.57	1.23-2.00	
Seldom	54	873	927	6	44	2558	2602	2	1.00		
Total	434	5229	5663	8	612	19 077	19 689	3			
Stress											
Unbearable	14	269	283	5	5	483	488	1	0.59	0.37-0.94	
More than average	81	1060	1141	7	87	2985	3072	3	1.05	0.88-1.25	
Some	326	3988	4314	8	387	11 963	12 350	3	1.00		
No stress	185	1680	1865	10	226	5246	5472	4	1.07	0.94-1.22	
Total	606	6997	7603	8	705	20 677	21 382	3			
Body mass index (kg/m2) c										
	
Less than 25	181	1904	2085	9	340	10 218	10 558	3	1.00		
25-30	319	3248	3567	9	310	8063	8373	4	1.15	1.02-1.29	
Over 30	100	1822	1922	5	59	2289	2348	3	0.77	0.64-0.93	
Total	600	6974	7574	8	709	20 570	21 279	3			
a Odds ratio (OR) and 95% confidence limits (CL) adjusted for age, subjects with and without cardiovascular disease combined.
b % of total in the category.
c Self-reported height and weight.
