# Supplementary material for: Use of plant stanol ester margarine among persons with and without cardiovascular disease: Early phases of the adoption of a functional food in Finland
Source: Nutr J. 2005 Jun 1;4:20. doi: 10.1186/1475-2891-4-20 (PMC1177987; doi:10.1186/1475-2891-4-20)
Supplement: Additional File 5 — Self-perceived health and diet among 35–84 year-old users and nonusers of plant stanol ester margarine. (Table 5) [file 1475-2891-4-20-S5.rtf]

Table 5. Self-perceived health and diet among 35-84 year-old users and nonusers of plant stanol ester margarine.

Characteristics	Subjects with cardiovascular disease	Subjects without cardiovascular disease	Users versus nonusers a	
	Users	Nonusers	Total	User	Users	Nonusers	Total	User		
	N	N	N	% b	N	N	N	% b	OR	CL (95%)	
Self-perceived health											
Good	225	2204	2429	9	441	12 803	13 244	3	1.57	1.29-1.91	
Average	285	3247	3532	8	232	6287	6519	4	1.50	1.24-1.83	
Poor	99	1630	1729	6	36	1628	1664	2	1.00		
Total	609	7081	7690	8	709	20 718	21 427	3			
Daily amount of spread on bread c										
	
Less than 20 g	89	913	1002	9	109	2419	2528	4	1.00		
20-25 g	32	362	394	8	42	852	894	5	0.96	0.73-1.27	
More than 25 g	48	494	542	9	43	1233	1276	3	0.81	0.62-1.04	
Total	169	1769	1938	9	194	4504	4698	4			
Use of alcohol d											
No units	113	1222	1335	8	113	3940	4053	3	1.00		
1-5 units	118	1307	1425	8	196	5169	5365	4	1.23	1.03-1.47	
6 units or more	116	1266	1382	8	197	4878	5075	4	1.50	1.25-1.79	
Total	347	3795	4142	8	506	13 987	14 493	3			
Diet e											
Healthy	398	3436	3834	10	513	10 952	11 465	4	2.94	2.56-3.37	
Unhealthy	130	2888	3018	4	156	8648	8804	2	1.00		
Total	528	6324	6852	8	669	19 600	20 269	3			
a Odds ratio (OR) and 95% confidence limits (CL) adjusted for age, subjects with and without cardiovascular disease combined.
b % of total in the category.
c Only Finrisk 1997 Survey.
d Number of alcoholic units (12cl wine, 4cl spirits or one 33cl bottle of beer, long-drink or cider) during past 7 days.
e Healthy diet includes at least three of following: daily use of vegetables, avoidance of fatty milk, use of margarine or no spread on bread, use of bread more than five slices per day.
