# Supplementary material for: Use of plant stanol ester margarine among persons with and without cardiovascular disease: Early phases of the adoption of a functional food in Finland
Source: Nutr J. 2005 Jun 1;4:20. doi: 10.1186/1475-2891-4-20 (PMC1177987; doi:10.1186/1475-2891-4-20)
Supplement: Additional File 6 — Medication among 35–84 year-old users and nonusers of plant stanol ester margarine (Table 6) [file 1475-2891-4-20-S6.rtf]

Table 6. Medication among 35-84 year-old users and nonusers of plant stanol ester margarine.

Characteristics	Subjects with cardiovascular disease	Subjects without cardiovascular disease	Users versus nonusers a	
	Users	Nonusers	Total	User	Users	Nonusers	Total	User		
	N	N	N	% b	N	N	N	% b	OR	CL (95%)	
Current use of cholesterol lowering drugs c	52	169	221	24	20	49	69	29	4.31	3.19-5.84	
Cholesterol lowering drugs d	128	504	632	20	73	190	263	28	6.25	5.24-7.46	
Hypertensive drugs ever c	114	1046	1160	10	12	168	180	7	1.80	1.30-2.48	
Hypertensive drugs d	295	3486	3781	8	23	209	232	10	1.67	1.44-1.94	
Current use of diabetic drugs c	15	124	139	11	4	63	67	6	2.80	1.14-6.87	
Painkiller d	227	3209	3436	7	225	7738	7963	3	0.80	0.72-0.90	
Sleeping pills d	81	942	1023	8	52	1058	1110	5	0.98	0.81-1.19	
Sedatives d	59	602	661	9	33	967	1000	3	1.08	0.86-1.34	
Antidepressants c, d	9	93	102	9	4	172	176	2	0.76	0.43-1.34	
Asthma drugs c, d	11	143	154	7	8	193	201	4	0.80	0.50-1.29	
Hay fever drugs c, d	6	40	46	13	4	82	86	5	1.38	0.71-2.67	
Acetylsalicylic acid for AMI prevention c, d	81	403	484	17	22	162	184	12	2.70	2.07-3.52	
Blood anticoagulants c, d	15	126	141	11	1	19	20	5	1.17	0.68-2.00
	
Cough syrup d	44	548	592	7	22	959	981	2	0.91	0.70-1.18	
Cardiovascular disease drugs d, e	87	913	1000	9	15	126	141	11	1.50	1.15-1.96	
a Odds ratio (OR) and 95% confidence limits (CL) adjusted for age, subjects with and without cardiovascular disease combined.
b % of total in the category.
c Only Finrisk 1997 Survey.
d During past 7 days.
e Only Elderly Health Behavior Survey 1997 and 1999.
